# Loss of ubiquitin E2 Ube2w rescues hypersensitivity of Rnf4 mutant cells to DNA damage

**DOI:** 10.1038/srep26178

**Published:** 2016-05-17

**Authors:** Jean-François Maure, Sandra C. Moser, Ellis G. Jaffray, Arno F. Alpi, Ronald T. Hay

**Affiliations:** 1 Centre for Gene Regulation and Expression, College of Life Sciences, University of Dundee, DD1 5EH, UK; 2 MRC Protein Phosphorylation and Ubiquitylation Unit, College of Life Sciences, University of Dundee, DD1 5EH, UK

## Abstract

SUMO and ubiquitin play important roles in the response of cells to DNA damage. These pathways are linked by the SUMO Targeted ubiquitin Ligase Rnf4 that catalyses transfer of ubiquitin from a ubiquitin loaded E2 conjugating enzyme to a polySUMO modified substrate. Rnf4 can functionally interact with multiple E2s, including Ube2w, *in vitro*. Chicken cells lacking Rnf4 are hypersensitive to hyroxyurea, DNA alkylating drugs and DNA crosslinking agents, but this sensitivity is suppressed by simultaneous depletion of Ube2w. Cells depleted of Ube2w alone are not hypersensitive to the same DNA damaging agents. Similar results were also obtained in human cells. These data indicate that Ube2w does not have an essential role in the DNA damage response, but is deleterious in the absence of Rnf4. Thus, although Rnf4 and Ube2w functionally interact *in vitro*, our genetic experiments indicate that in response to DNA damage Ube2w and Rnf4 function in distinct pathways.

The failure to properly correct DNA damage is thought to be a major driving force in the development of cancer and ageing. Familial backgrounds carrying mutations in genes involved in DNA damage repair pathways often produce offspring which develop syndromes with severe brain malformation and/or tumour predisposition^1^. The most common type of DNA lesions is the loss or modification of a single base or nucleotide which occurs at 10^4^–10^5^/cell per day^2^,^3^. Direct nucleotide modifications are repaired by Base Excision Repair (BER) and Nucleotide Excision Repair (NER). One of the most hazardous types of DNA damage is when both strands of the DNA double helix are broken. DNA double strand breaks are mostly processed by two repair pathways: homologous recombination (HR) and non-homologous end joining (NHEJ). NHEJ is the predominant repair pathway in human cells and represents the rejoining, by blunt end ligation, of the two double strand ends of DNA generated by the break. As the broken ends are susceptible to exonucleases the joining of damaged ends may lead to mutation^4^. The HR pathway operates in S/G2 phase of the cell cycle where it uses the unbroken replicated sister chromatids as a template to copy and repair the broken chromosome.

Interstrand Crosslinks (ICLs) in DNA are generated by cancer therapy drugs like cisplatin and mitomycin C (MMC). These drugs physically link the two DNA strands thereby interfering with gene transcription and DNA replication. This DNA damage is repaired by the ICL repair pathway which is achieved by combining activities from multiple repair pathways including helicase, nucleotide excision repair, homologous recombination and translesion synthesis (TLS)^5^,^6^. Post-translational modifications of repair proteins allow cells to tightly control the action and efficiency of repair machinery components to ensure efficient repair and avoid the propagation of damaged DNA.

The activity of DNA damage repair components has been shown to be under control of several types of post translational modification including phosphorylation and ubiquitination. Protein ubiquitination is critical for DNA double strand break (DSB) repair by homologous recombination^7^. Absence of protein polyubiquitination is observed after inactivation of several ubiquitin E3 ligases such as Rnf8, Rnf168 and Brca1 which correlates with a failure to properly repair the DSB by homologous recombination, ultimately leading to several human syndromes or cancer. Cells from Fanconi anemia (FA) patients are hypersensitive to ICL damage^8^,^9^. The FA core complex promotes monoubiquitination of FancI and FancD2 which facilitates ICL repair by recruitment of downstream factors like nucleases, to the site of DNA damage^10^,^11^,^12^,^13^,^14^,^15^.

Recently, Small Ubiquitin Modifier (SUMO) has been shown to play a role in the response of cells to DNA damage^16^,^17^,^18^,^19^. The core enzymatic cascade that mediates protein SUMOylation is conserved in all eukaryotes. In humans, there are three SUMO paralogues, SUMO1, SUMO2 and SUMO3 that can be linked to the side chains of lysine residues in target proteins. The SUMOylation consensus site ΨKx(D,E) (Ψ: hydrophobic amino acid, X: any aminoacid) is found in SUMO2/3 but not SUMO1 which allows the formation of chains of SUMO2/3 (polySUMO). SUMOylation is involved in for many cellular processes including protein stability, protein interaction, activity and localization^20^. Mutation or altered regulation of enzymes essential for protein SUMOylation have been implicated in cancer, heart disease, virus infection and diabetes^21^,^22^,^23^. Rnf4 is a RING type E3 ubiquitin ligase which contains four SUMO interaction motifs in its N-terminal region that allow the protein to interact with SUMO chains and drive their ubiquitination^24^. Recent work revealed that Rnf4 is essential for DNA damage repair of DSB by homologous recombination^25^,^26^. Together these observations suggest that Rnf4 is a key protein which allows the ubiquitination of SUMOylated protein targets after DNA damage and that ubiquitination and SUMOylation modification pathways are closely interconnected by Rnf4 to repair damaged DNA.

To promote ubiquitination upon DNA damage, the ubiquitin E3 ligase Rnf4 must associate with its cognate ubiquitin E2 conjugating enzyme. Ube2w is an unusual E2 enzyme that catalyses N-terminal monoubiquitination of target proteins *in vitro*^27^,^28^. Recent work revealed that Ube2w can functionally interact with Rnf4 to promote N-terminal mono-ubiquitination of SUMO chains *in vitro*^27^. Monoubiquitinated SUMO chains can be further modified by the heterodimeric E2 conjugating enzyme Ubc13/Uev1 to form K63 linked ubiquitin chains^27^. K63-ubiquitin chains formed by the Ubc13 complex are important for DNA damage repair by homologous recombination. In DNA damaged cells that are depleted of Rnf4, the levels of K63 ubiquitin chains are reduced^25^. Ube2w has also been shown to allow anchoring of K63 ubiquitin chains on internal lysine of Trim5α during restriction of HIV reverse transcription^29^. Together these observations suggest that Ube2w and Rnf4 may work together to promotes K63 ubiquitin chain formation upon DNA damage.

Here we show that cells lacking Rnf4 but not Ube2w are hypersensitive to replication stress, inter strand DNA crosslinking agents and DSB caused by γ-irradiation. In contrast, cells lacking Ube2w are not hypersensitive to the DNA damaging agents tested, but inactivation of Ube2w in ΔRNF4 mutant cells rescues the DNA damage sensitivity. These data indicate that Ube2w does not have an essential role in the DNA damage response, but is deleterious in the absence of Rnf4. Thus, while Rnf4 and Ube2w functionally interact *in vitro*, these genetic experiments suggest that in response to DNA damage Ube2w and Rnf4 function in distinct pathways.

## Results

### Ube2w inactivation suppresses ΔRNF4 hypersensitivity to DNA damage inducing drugs

In human cells, the role of Rnf4 in DSB repair by HR, NHEJ and ICL repair has been previously described^25^,^26^,^30^. In DT40 cells, a role for Rnf4 in DNA damage repair was observed in replication associated stress^25^. As an ubiquitin E3 ligase Rnf4 must work in DNA repair with the help of an ubiquitin E2. The ubiquitin E2 Ube2w has been shown to promote monoubiquitination of SUMO chains in an Rnf4 dependent manner *in vitro*^27^.

**Figure 1:**
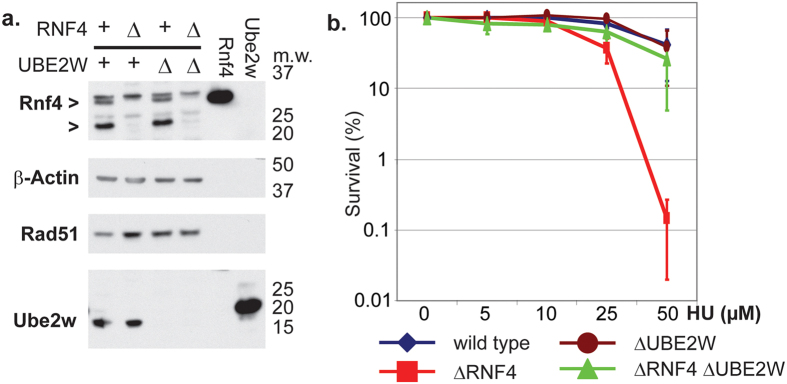
Ube2w inactivation rescues ΔRNF4 replication stress hypersensitivity. (**a**) Whole cells extracts of Chicken DT40 wild type cells and cells deficient for Rnf4 (ΔRNF4), Ube2w (ΔUBE2W) and Rnf4 and Ube2w (ΔRNF4, ΔUBE2W) were analysed by western blotting using the indicated antibodies. 3 ng of recombinantly expressed rat Rnf4 and Human Ube2w isoform1 protein were used as a control. Molecular weight markers (kDa) are indicated on the right. (**b**) Wild type cells and cells deficient for ΔRNF4; ΔUBE2W and ΔRNF4, ΔUBE2W were subjected to replication stress by HU. The concentration of HU is indicated on the X-axis (μM). Effect on each cell line is indicated by the percentage of colony formation on the Y-axis (logarithmic scale). Error bars represent two standard deviations from the mean (2SD).

To study the role of Ube2w and Rnf4 in DNA damage repair at the genetic level, we created chicken DT40 cell lines deleted for RNF4 (ΔRNF4); UBE2W (ΔUBE2W) and the double deletion ΔRNF4 ΔUBE2W (Fig. 1a). The three cell lines were viable and proliferated at similar rates as the wild type cell line. We subjected each cell line to DNA damage inducing drugs and measured the cell survival rate by colony formation assay. As observed before, ΔRNF4 cells were hypersensitive to replication stress caused by hydroxyurea (HU) exposure (Fig. 1b). ΔUBE2W mutants showed no sensitivity to HU induced replication stress (Fig. 1b). Surprisingly, we observed that the double deletion cell line of ΔRNF4 ΔUBE2W was not hypersensitive to HU induced replication stress (Fig. 1b). These results showed that inactivation of Ube2w in a ΔRNF4 background rescued the HU-induced replication stress hypersensitivity observed in ΔRNF4 mutants, thus suggesting that Ube2w is deleterious in the absence of Rnf4 and that Ube2w and Rnf4 function in distinct pathways of DNA damage repair.

**Figure 2:**
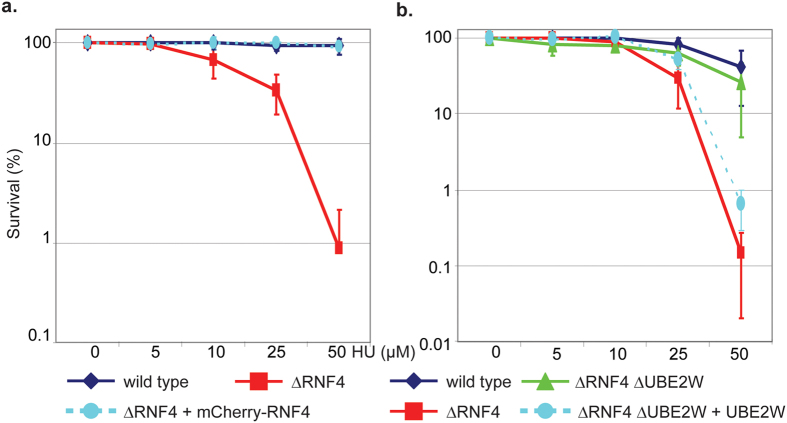
Complementation of ΔRNF4 and ΔRNF4 ΔUBE2W mutant defects. (**a**) Wild type cells; ΔRNF4 and ΔRNF4 cells complemented with chicken mCherry-Rnf4 as described in Fig. 1 were subjected to colony survival assay after treatment with the indicated concentration of HU. (**b**) Wild type cells; ΔRNF4; ΔRNF4 ΔUBE2W and ΔRNF4 ΔUBE2W complemented with Human Ube2w isoform2 were subjected to colony survival assay with the indicated concentrations of HU. Data represented as indicated: Wild type (Blue losange), ΔRNF4 (red square), ΔRNF4 ΔUBE2W (green triangle), rescue clone (circle opal). Error bars represent 2 SD.

To confirm that the hypersensitivity to HU induced replication stress was due to Rnf4 deficiency and not caused by secondary mutation we expressed mCherry-Rnf4 in the ΔRNF4 cell line. ΔRNF4 hypersensitivity to HU was entirely complemented by the expression of chicken mCherry-Rnf4 (Fig. 2a and supplementary Fig. S1a). Similarly, to confirm the specific role of Ube2w in ΔRNF4 ΔUBE2W double mutant cells, we ectopically expressed human E2 Ube2w isoform 2 in these cells (Fig. 2b and supplementary Fig. S1c) and demonstrated that they regained the HU hypersensitivity of DT40 ΔRNF4 cells. ΔUBE2W mutant cells are not sensitive to HU, but overexpression of human E2 Ube2w in these cells induced a mild level of sensitivity to HU induced replication stress (supplementary Fig. S1b). We noted that tagged forms of Ube2w were unable to restore the HU hypersensitivity of ΔRNF4 ΔUBE2W cells suggesting that tagging Ube2w renders the protein inactive *in vivo* (supplementary Fig. S1g,h).

**Figure 3:**
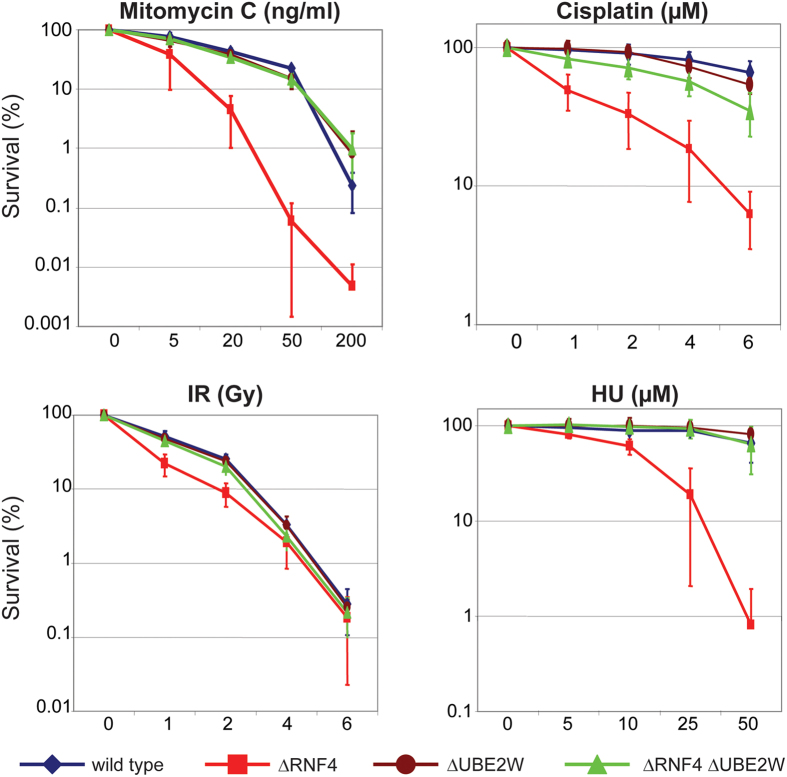
Ube2w inactivation suppressed ΔRNF4 DNA damaged hypersensitivity. Wild type cells and cells deficient for ΔRNF4, ΔUBE2W and ΔRNF4 ΔUBE2W were subjected to DNA damaged induced stress by mitomycin C, cisplatin, γ-irradiation, HU as indicated. X-axis indicated the increase range of concentration for each drug. Effect on each cell line is indicated of the percentage of colony formation on the Y-axis (logarithmic scale). DATA represented as indicated: Wild type (Blue losange), ΔRNF4 (red square), ΔUBE2W (brown circle), ΔRNF4 ΔUBE2W (green triangle). Error bars represent 2 SD.

We next tested the sensitivity of ΔRNF4 mutant cells to other types of DNA damage. In addition to HU, ΔRNF4 cells are hypersensitive to ionising radiation, MMC and cisplatin (Fig. 3) and display mildly increased sensitivity to the DNA alkylating agent methyl methanesulfonate (MMS) (supplementary Fig. S2a). In each case the observed hypersensitivity is lost in ΔRNF4 ΔUBE2W cells (Fig. 3). Although ΔRNF4 cells are hypersensitive to the DNA polymerase inhibitor aphidicolin, this hypersensitivity was not rescued in ΔRNF4 ΔUBE2W cells (supplementary Fig. S2a). As observed for HU (Fig. 1) ΔUBE2W mutant cells are not hypersensitive to any of these agents (Fig. 3, supplementary Fig. S2a), although they do display a mild hypersensitivity to etoposide (supplementary Fig. S2a). Thus Ube2w has only a limited non-redundant role in the DNA damage response.

### Prolonged FancD2/I monoubiquitination induced by MMC in the absence of by Rnf4 is rescued by UBE2W inactivation

Cisplatin and MMC generate interstrand ICLs in DNA that are repaired by the ICL repair pathway. The ΔRNF4 mutant cell line is hypersensitive to the ICL inducing drugs MMC and cisplatin: at 50 ng/ml MMC, ΔRNF4 cells are greater than 200 fold more sensitive than wild type cells (Fig. 3). This extreme sensitivity to DNA cross linking agents is also observed in DT40 cells with mutations in FA pathway components^5^. Thus to establish if the FA pathway is functional in ΔRNF4 cells we determined the levels of monoubiquitinated FancI and FancD2 upon MMC treatment.

**Figure 4:**
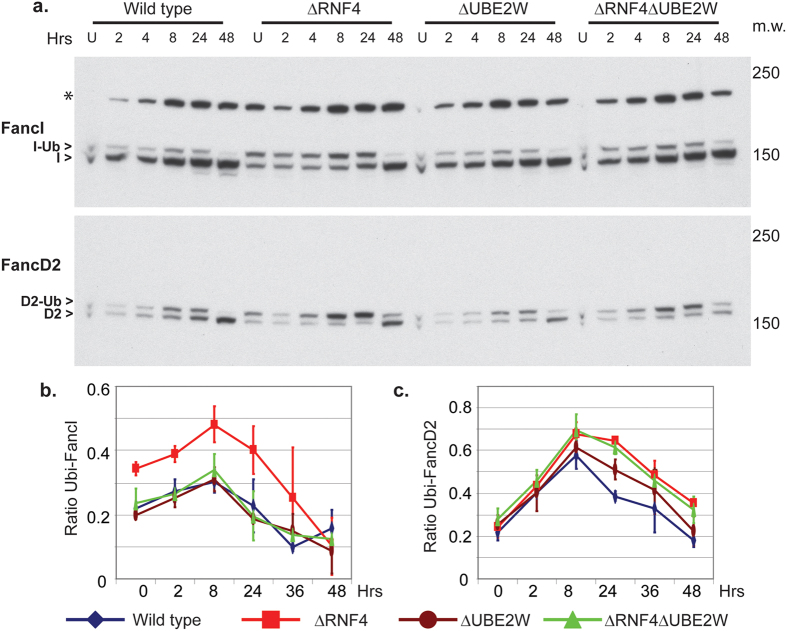
Increased and prolonged FancD2/I monoubiquitination induced by MMC in ΔRNF4 is rescued by UBE2W inactivation. Wild type cells and cells deficient for ΔRNF4, ΔUBE2W and ΔRNF4 ΔUBE2W were treated with MMC (100ng/ml) for 1H. (**a**) Proteins were analysed by western blot using antibodies against FancI (top panel) and FancD2 (bottom panel). The recovery time after MMC treatment of each protein extraction is indicated on each lane of the top panel in hours. Non-ubiquitinated and mono-ubiquitinated form of FancI and FancD2 are indicated by an arrows head on the left inside (respectively: I>, D2> and I-Ub>; D2-Ub>). Asterisk indicates non specific band (*). Molecular weight markers are indicated on the right inside (kDa). (**b,c**) quantification of the ratio of ubiquitinated protein to total protein has been determined by three independent LiCOR experiment for FancI and FancD2. LiCOR images can be seen in supplementary Fig. S3.

In response to a 60 minutes MMC treatment, the levels of both monoubiquinated FancI and FancD2 increase in all cell lines, reaching peak levels around 8–24 H after treatment (Fig. 4a). This indicates that the FA pathway is activated in all of the mutant cell lines. To compare the efficiency and timing of ubiquitin modification of FancI and FancD2 in response to MMC in wild type and ΔRNF4, ΔUBE2W and ΔRNF4 ΔUBE2W cells, extracts were analysed by quantitative Western blotting (Fig. 4b,c and supplementary Fig. S3). It is known that 24–48 H after treatment the decreased levels of monoubiquitinated FancI and FancD2 are a reflection of active ICL repair. Consistent with recent observations in human cells^31^ the monoubiquitinated form of FancI accumulates to a higher level in response to MMC treatment in ΔRNF4 cells compared to wild type cells (Fig. 4b,c and supplementary Fig. S3). Levels of monoubiquitinated FancI were similar in wild type, ΔUBE2W and ΔRNF4 ΔUBE2W cells, indicating that the increased accumulation of monoubiquitinated FancI in ΔRNF4 cells was abrogated by codepletion of Ube2w (Fig. 4b,c and supplementary Fig. S3). In untreated cells the levels of monoubiquitinated FancD2 were similar in all cell types as was the rate of accumulation of monoubiquitinated FancD2 in response to MMC (Fig. 4b,c and supplementary Fig. S3).

### Prolonged DNA damage induced foci formation in ΔRNF4 cells is suppressed by UBE2W inactivation

**Figure 5:**
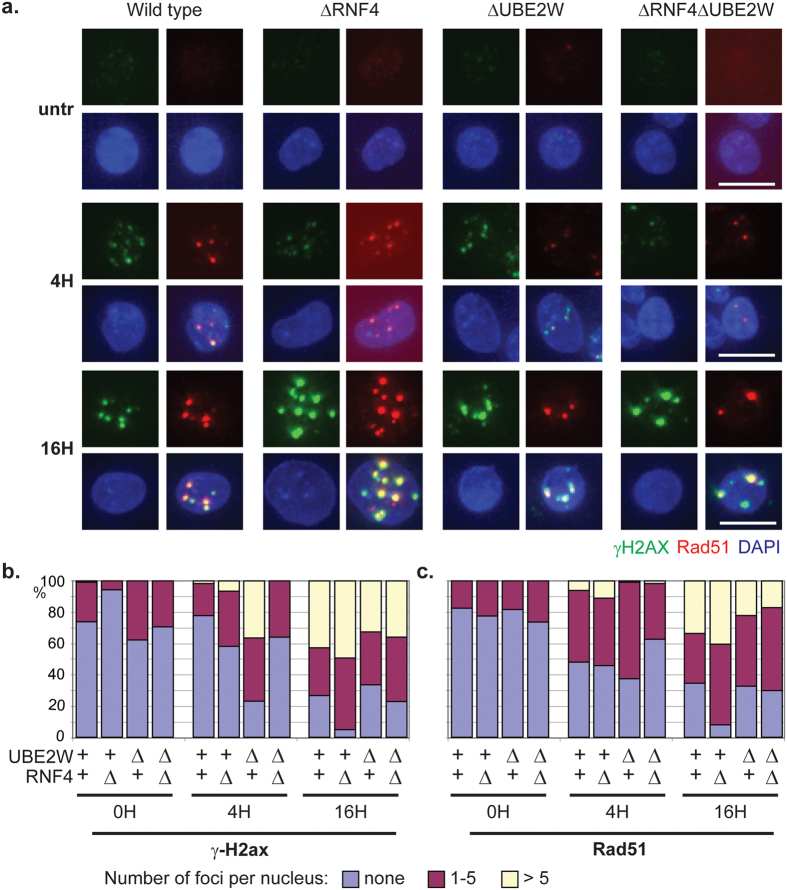
Prolonged DNA damage induced foci formation in ΔRNF4 cells is suppressed by UBE2W inactivation. After treatments with or without MMC (50ng/ml) for 1H, cells were allowed to recover for 4H or 16H. (**a**) For each indicated cell line, the formation of MMC induced foci for γ-H2ax (green) and Rad51 (red) was analysed by immunostaining. Chromatin was visualized by DAPI (Blue). Representative images are shown for each staining and merged images. White bar: 10 um. (**b,c**) percentage of cell containing no focus; 1–5 foci; >5 foci are represented for each conditions respectively in blue, red and yellow. (**a**) γ-H2ax. (**b**) Rad51 quantification. More than 100 cells have been counted for each condition.

Sustained monoubiquitination of FancI in response to MMC in ΔRNF4 cells compared to wild type levels of monoubiquitinated FancI in ΔRNF4 ΔUBE2W cells suggests that a late step in the repair pathway is defective in the absence of Rnf4 when Ube2w is present, but this pathway is restored when Rnf4 and Ube2w are codepleted. Following activation of the FA pathway, the assembly and disassembly of Rad51 filaments is a key step in the repair of ICL DNA damage. We therefore assessed the formation of Rad51 foci as readout for a late step in the repair of damaged DNA. As an early marker of DNA damage we co-stained with γ-H2ax. Cells were either untreated or treated with MMC for 60 minutes and then fixed and stained after 4 or 16H. Quantification of the number of γ-H2ax and Rad51 foci per nucleus over time provides a read out of DNA repair. Untreated wild type and mutant cells exhibit only a few γ-H2ax and Rad51 foci (Fig. 4a–c), but after MMC treatment, all cells lines displayed an increased number of γ-H2ax and Rad51 foci per nucleus. 16H after DNA damage, more than a third of the wild type, ΔUBE2W and ΔRNF4 ΔUBE2W cells had no γ-H2ax or Rad51 foci suggesting that the DNA damage is repaired in these cells (Fig. 5a–c and supplementary Fig. S4). However, in ΔRNF4 cells 16H after recovery, more than 90% of cells show a large number of large γ-H2ax and Rad51 foci, suggesting that ICL DNA repair foci are not resolved efficiently in ΔRNF4 cells. ΔRNF4 ΔUBE2W cells have a similar number of γ-H2ax and Rad51 foci per nucleus as wild type cells indicating that the repair defect in ΔRNF4 cells is rescued by inactivation of E2 Ube2w. This indicates that in the absence of Rnf4, the presence of Ube2w results in a late stage defect in the repair of damaged DNA.

### The deleterious effect of Ube2w in the absence of Rnf4 is conserved in human cells

**Figure 6:**
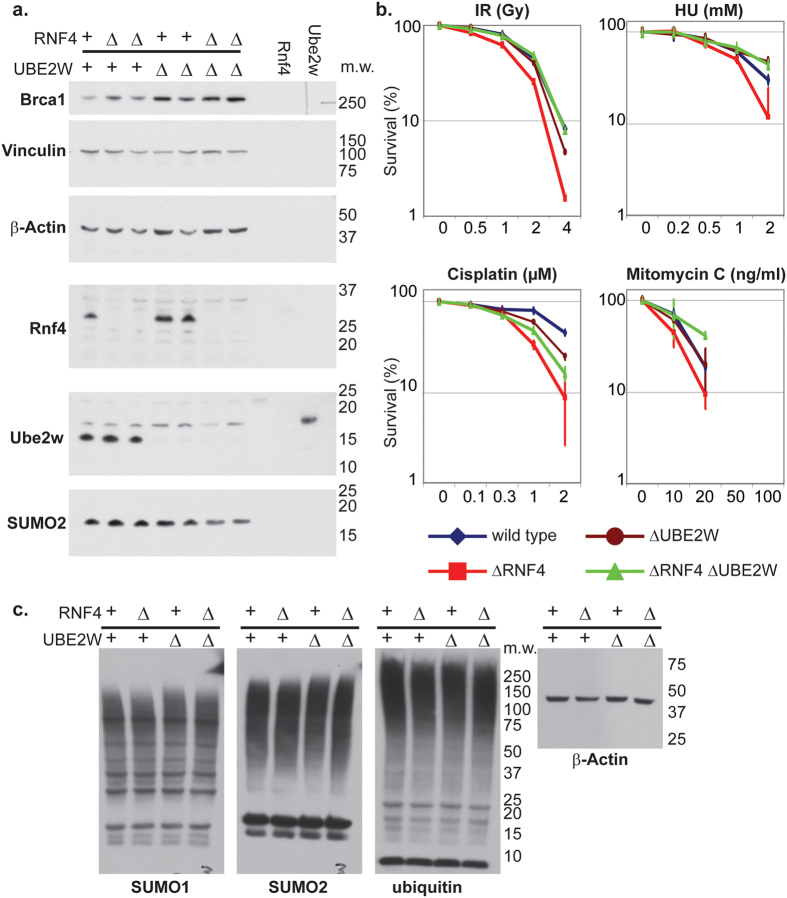
Suppression of ΔRNF4 DNA damage hypersensitivity by ΔUBE2W is conserved in Human. (**a,c**) Whole cells extracts of Human HCT116 wild type cells and cells deficient for Rnf4 (ΔRNF4), Ube2w (ΔUBE2W) and Rnf4 and Ube2w (ΔRNF4 ΔUBE2W) were analysed by Western blotting using the indicated antibodies. 3 ng of recombinantly expressed rat Rnf4 and Human Ube2w isoform1 protein were used as a control. Molecular weight marker is indicated on the right inside (kDa). (**b**) Wild type cells and cells deficient for ΔRNF4; ΔUBE2W and ΔRNF4, ΔUBE2W were subjected to cisplatin, mitomycin C, γ-irradiation and replication stress by HU. The concentration of HU is indicated on the X-axis. Effect on each cell line is indicated of the percentage of colony formation on the Y-axis (logarithmic scale). Data represented as indicated: Wild type (Blue losange), ΔRNF4 (red square), ΔUBE2W (brown circle), ΔRNF4 ΔUBE2W (green triangle). Error bars represent 2 SD.

To analyse the genetic interactions between Ube2w and Rnf4 in the DNA damage response of human cells CRISPR/Cas9 technology was used to generate ΔRNF4, ΔUBE2W and ΔRNF4 ΔUBE2W cells (Fig. 6a) These cell lines were treated with DNA damaging drugs and cell survival determined by clonogenic survival assays. ΔRNF4 cells were hypersensitive to hydroxyurea (Fig. 6b), although the defects observed were less pronounced than in chicken cells. As observed in chicken cells, the defect caused by depletion of Rnf4 is rescued by codepletion of Ube2w. ΔUBE2W cells were not sensitised to DNA damage (Fig. 6b). These results suggest that the deleterious effect of Ube2w in the absence of Rnf4 is conserved in human cells and that Ube2w and Rnf4 function in distinct pathways of DNA repair.

## Discussion

The role of the ubiquitin E2 conjugating enzyme Ube2w has been rather elusive. While the ubiquitin E2 conjugating enzyme Ube2T has been shown to functionally interacts with the FancL E3 ligase to mono-ubiquitinate FancI and FancD2 in response to ICL^32^,^33^,Ube2w was reported to promote monoubiquitination of FancD2 upon UV damage^34^. An *in vitro* study showed that Ube2w can promote monoubiquitination on FancD2 also in the absence of E3 ligase FancL^11^. Critically, *in vivo* studies of Ube2w/Ubc16 in several organisms failed to identify a role in DNA damage^34^,^35^,^36^. In agreement with these observations we have found that ΔUBE2W cells are not sensitised to any of the DNA damaging agents tested. This is also consistent with data from a high throughput siRNA screen assessing the role of all known E2 conjugating enzymes in the DNA damage response. Knockdown of Ube2w did not lead to a major defect in repair efficiency and did not alter the number of foci per cell staining with 53BP1 and γ-H2ax, although there was a 50% reduction in the number of foci per cell that stained with the anti-ubiquitin antibody FK2^37^. Using a genetic approach in chicken DT40 cells we could show that in the absence of Rnf4 the presence of Ube2w has a deleterious effect on cell survival in response to a range of DNA damaging agents. The precise molecular explanation for this observation has not been established but one possibility is that in combination with an E3 ligase other than Rnf4, Ube2w monoubiquitinates a substrate or substrates in response to DNA damage that acts to prime the substrate for the Rnf4 mediated synthesis of K63 ubiquitin chains that may create a platform for the action of effector molecules. In the absence of Rnf4 the normally transient, monoubiquitinated intermediate created by Ube2w would accumulate and its failure to be further ubiquitinated might block a downstream step in DNA repair. As ΔUBE2W cells are not hypersensitive to DNA damaging agents the monoubiquitination mediated by Ube2w must be redundant and in the absence of Ube2w this modification could be carried out by another E2 ubiquitin conjugating enzyme. In fact it is known that Ube2w can work with multiple E3 ligases and could generate monoubiquitinated substrates in combination with Brca1^38^. Likewise Rnf4 has been shown to functionally interact with multiple ubiquitin E2 conjugating enzymes including Ube2w, Ubc13/Uev1 and UbcH5^27^. Identification of targets for Ube2w mediated monoubiquitination in cells lacking Rnf4 is challenging but would represent a major step forward in understanding the deleterious role of Ube2w in the absence of Rnf4. Hypersensitivity to cisplatin in Fanconi anemia mutant ceIls caused by a failure to repair interstrand crosslinks can be suppressed by inactivating the NHEJ pathway^39^,^40^. Therefore the suppression of DNA damage hypersensitivity in ΔRNF4 by the inactivation of Ube2w could be explained if Ube2w would be a component of NHEJ pathway. SUMO modification of FancI and FancD2 has been shown to allow recruitment of Rnf4, leading to polyubiquitination of FancI and FancD2 and their removal by the Dvc1-p97 segregase^31^. Early work suggested that Ube2w can promote monoubiquitination of FancD2 upon UV treatment and Ube2w interact with FancL at DNA damage repair foci^34^. However, these experiments have been carried out by using tagged versions of Ube2w which in our hands were non functional *in vivo*^34^. We observed an increase in monoubiquitination of FancI/D2 upon MMC treatment in ΔRNF4 cells which is suppressed by the inactivation of ΔUBE2W. The role of Ube2w in this process is unclear but taken together these observations suggest that the control of post translational modification of FancI/D2 is a critical step controlled by Rnf4 and Ube2w.

It is possible that the increase in monoubiquitination of FancI/D2 observed in ΔRNF4 is due to the lack of efficient DNA repair in these cells. Indeed, we could observe in ΔRNF4 cells upon MMC treatment an increased level of Rad51 foci formation which is an indication of repair delay or failure after ICL damage. However, Rnf4 inactivation in human cells by siRNA leads to a reduction of Rad51 recruitment after DNA double strand break^25^,^26^. This discrepancy in observations can be explained by differences in the regulation of DNA repair pathways in different cell lines. Chicken DT40 cells have a rapid cell cycle with an extended S-phase that favours homologous recombination, whereas the predominant repair pathway in most human cells is NHEJ. Thus Rnf4 may act on several targets at different stages on the same pathway as suggested by Yin *et al.*^25^. We observed an increased recruitment of Rad51 after ICL damage which is a different type of DNA damage from that studied in Yin *et al.*^25^. It is possible that Rad51 has a different role and regulation in these two types of DNA damage. A recent study of a Rad51 dominant mutant revealed its function in ICL repair independently of HR repair^41^. This suggests that Rad51 and its regulators are potential targets for Rnf4 and Ube2w to control DNA repair.

Ube2w is ubiquitously expressed in tissues and cells at variable levels^35^,^36^. The highest expression level of Ube2w is found in prostate, breast and lung cell lines^35^,^36^. This enzyme has been identified only in higher eukaryotes^34^. In human and mouse, three isoforms have been described with variable N-terminal and C-terminal extensions due to alternative splicing (NCBI). A recent study has shown that the flexible C-terminus of Ube2w is involved in dimerisation and substrate specificty^42^,^43^. It is thus possible that different isoforms of Ube2w have different activities although this has not been tested. Ube2w isoform one mediates N-terminal monoubiquitination *in vitro*^27^,^28^. However, in cells, the N-terminus of most proteins is processed and post translationally acetylated. As this would block N-terminal ubiquitination by Ube2w, substrates for N-terminal ubiquitination by Ube2w would have to be created by proteolitic cleavage or *de novo* protein synthesis. A recent study shows that in the case of Trim5α, Ube2w modifies a lysine side chain to prime the synthesis of K63-linked ubiquitin chain *in vivo* rather than promoting N-terminal mono-ubiquitination^29^. Identifying the target and each Ube2w isoform activity upon DNA damage treatment is a crucial but challenging step in order to understand the molecular mode of action of Ube2w.

In summary this work reveals for the first time that depletion of Rnf4 unmasks a deleterious action of Ube2w in DNA damage repair pathway after ICL, alkylated DNA damage and replication stress and suggests that Ube2w and Rnf4 function in distinct pathways of DNA repair.

## Materials and Methods

### Generation of RNF4^−/−^ and UBE2W-/-/- cell lines

The scheme for the generation of the *UBE2W* gene disruption is outlined in (supplementary Fig. S5). Note, in DT40 cells the *UBE2W* locus is on the trisomic chromosome II. The targeting construct for *UBE2W* disruption was generated by amplifying the 5′ homology arm using DT40 genomic DNA as a template and the PCR primer pair 5′ GCAAAATGAT CCACCTCCCG GAATGAC and 5′ GGAATATTGT CACCAGTAAA CATGACC, and cloned into pCR2.1 using the TOPO TA cloning kit (Invitrogen). The 5′arm was recovered as a NotI/BamHI fragment and cloned into a pBluescript vector to generate pBS-UBE2W5′arm. The 3′ homology arm was amplified using the primer pair 5′ GTAAGGCAGG ATGGGAGGGA CAGAGTTAG and 5′ GCAGCAAGTT CAGTTATATC ACTGCCATCC, cloned into pCR2.1, recovered as BamHI/EcoRV fragment and cloned into pBS-UBE2W5′arm. The puromycin (first allele), blasticidin (second allele) and histidinol (third allele) resistance cassettes were inserted into the BamHI restriction site. Targeted integrations were detected by Southern blot analysis of BamHI/KpnI-digested genomic DNA. Generation of RNF4 knock out has been described previously^25^.

### CRISPR/Cas9 knock out

Gene knock out in human cell using CRISPR/Cas9 technology has been described previously^44^,^45^. Shortly, humanized Cas9 wild type (Addgene 42229/ pX260) or nickase Cas9-D10A (Addgene 42333/ pX334) combined with gRNA targetting vector were transfected into cells using GeneJuice (Millipore). Each targeting protospacer for human RNF4 and UBE2W were introduced by direct PCR mutagenesis into pA608 (pA608 is modified version of Addgene 41824 guide RNA cloning vector). The sequence used to target Human RNF4 gene is “gacgctttctctgagtagca” (pA622) and “gctactcagagaaagcgtcg” (pA624). The sequence used to target Human UBE2W gene is “gttccatcatggcgtcaatgc” (pA629).

### Plasmids for complementation

*pCMV-mCherry-ggRNF4 (pA626):* Gallus gallus Rnf4 (NP_001012907.1, NCBI) containing the polymorphism (G169 >D169) was cloned from DT40 cDNA into a modified version of pCMV-mCherry-C1 (Clontech) were a chicken hygromycin resistance cassette was inserted into BamH1 site.

### pCAG-hUBE2W isoF2 (pA670)

Human Ube2w (NCBI NP_060769.4/ (M30-C151) was subcloned into home made vector under CAG promoter containing chicken Hygromycin resistance cassette.

### pCMV-hUBE2W isoF1 (pA631)

Human Ube2w (NCBI NP_001001481.2/ (M30-C162) was subcloned into pCMV-EGFP/G418 vector (clonTech).

### Expression of recombinant protein

Gallus gallus Rnf4 (NP_001012907.1, NCBI) containing the polymorphism (G169 >D169) was cloned from DT40 cDNA into pLou3 in order to express HIS6-MBP-TEV-ggRnf4 (pA612). Technical purification detail has been described previously^24^. Briefly, ggRnf4 was expressed in BL21 Rosetta 2 and purified on Nickel column. The tag was cleaved off by TEV protease. *Rattus norvegicus* Rnf4 and *homo sapiens* Ube2w purification were described before^24^,^27^.

### Antibody affinity purification

The procedure was described previously^24^. Briefly, 30mg of dialysed recombinant protein untagged HsUbe2w (or ggRnf4) were covalently bound to activated NHS sepharose beads. 5ml of each sheep serum pass onto the affinity column. Bound antibodies were eluted with 0.1M glycine.

### Cell culture

DT40 cell transfections and culture were described previously^25^,^46^,^47^. Antibiotics used were Puromycin (0.5 μg/ml); Hygromycin (2 mg/ml, Roche 10843555001); Blasticidin (20 μg/ml, Melford B1105); Histidinol (1 mg/ml, SIGMA H6647-3G), G418 (2 mg/ml, Calbiochem 345812) in RPMI-1640 (Gibco/Invitrogen 3187-025)

### Protein Extraction for western blotting and LiCOR

Twenty million cells of each cell line were collected by centrifugation and wash with PBS 1X. Cells were resuspended in Lysis buffer (20 mM Tris pH7.5; 150 mM NaCl; 0.5% NP40; 50 mM β-glycerophosphate; 50 mM NaF; 2 mM NaVo4, Complete protease inhibitor Roche tablet) for 10 minutes. Lysates were centrifuged for ten minutes at 14000 g/4 °C. Supernatants were collected and sample buffer added prior to boiling. 50 μg of protein were separating by SDS-PAGE. Proteins were transferred onto PVDF membrane (Immubilon-P; 0.45 μm) for western blot detection. Proteins were transferred onto nitrocellulose membrane for LiCOR detection. Antibodies uses in this study are described in Supplementary Table1. FancI/D2 protein extraction and detection were described previously in^46^.

### Immunofluorescence

DT40 cells were attached for 20 minutes onto coverslip pretreated with 0.2% Concanavalin A (SIGMA: C7275). Cells were pre-extracted with 0.2% PBS triton for two minutes at 37 °C before seven minutes of fixation with 4% PFA. Cells were then processes for immunofluorescence as described previously in^25^.

## Additional Information

**How to cite this article**: Maure, J.-F. *et al.* Loss of ubiquitin E2 Ube2w rescues hypersensitivity of Rnf4 mutant cells to DNA damage. *Sci. Rep.* **6**, 26178; doi: 10.1038/srep26178 (2016).

## Supplementary Material

Supplementary Information
