# Supplementary material for: Loss of ubiquitin E2 Ube2w rescues hypersensitivity of Rnf4 mutant cells to DNA damage
Source: Sci Rep. 2016 May 17;6:26178. doi: 10.1038/srep26178 (PMC4868978; doi:10.1038/srep26178)
Supplement: Supplementary Information [file srep26178-s1.doc]

**Loss of ubiquitin E2 Ube2w rescues hypersensitivity of Rnf4 mutant cells to DNA damage**

Maure Jean-François^1^, Moser Sandra C.^1^, Jaffray Ellis G.^1^, Alpi F. Arno^2^, Hay Ronald T.^1^*

^1^ Centre for Gene Regulation and Expression, College of Life Sciences, University of Dundee, DD1 5EH, UK

^2^ Department of MRC Protein Phosphorylation and Ubiquitylation Unit, College of Life Sciences, University of Dundee, DD1 5EH, UK

* Corresponding author: [R.T.Hay@dundee.ac.uk](mailto:R.T.Hay@dundee.ac.uk)


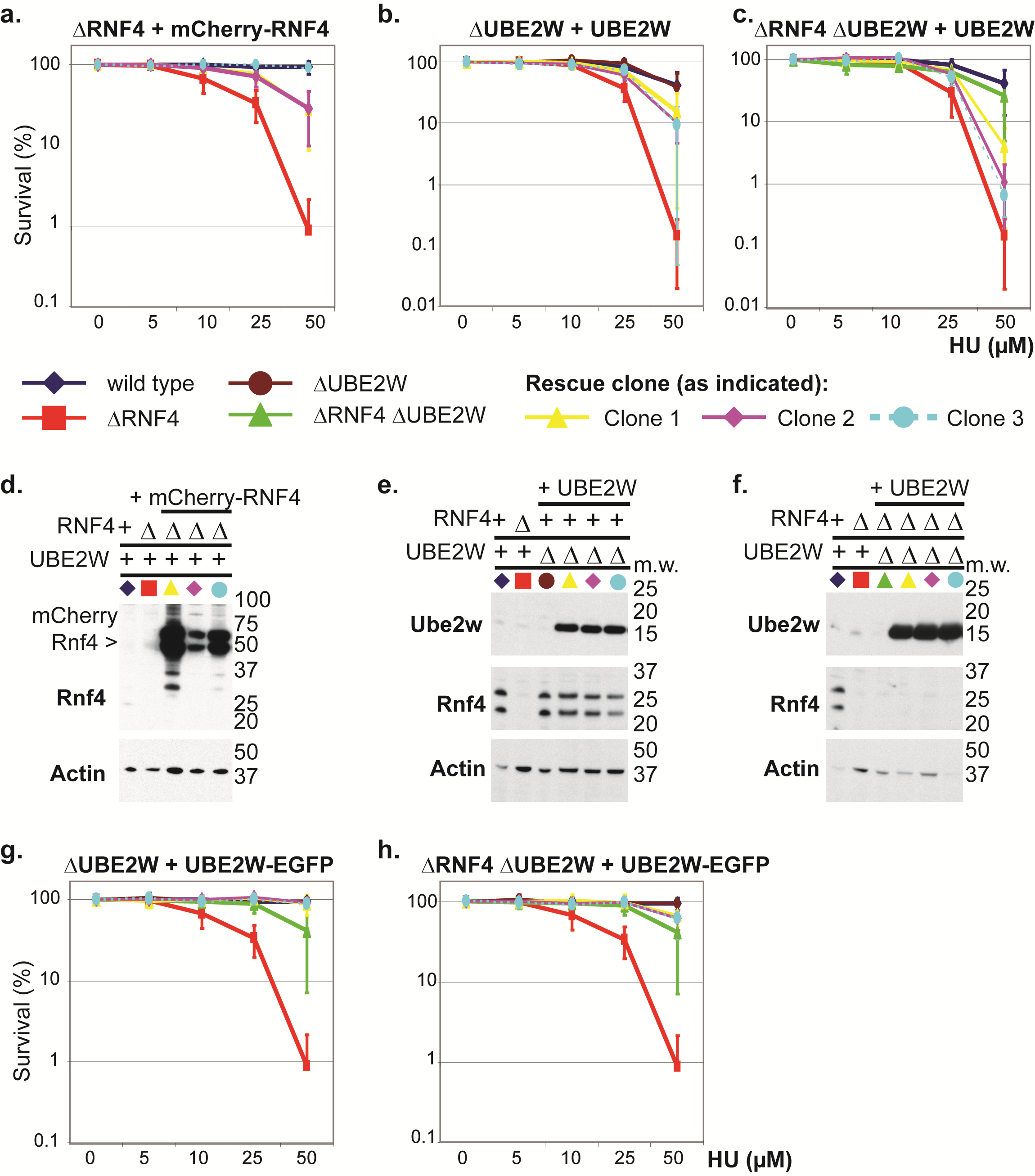


**Supplementary Figure 1: Complementation of ΔRNF4; Δube2W and ΔRNF4 ΔUBE2W defect**

Mutants complementation was determined by colony survival assay as described in (Fig. 1). **(a)** Wild type cells; ΔRNF4 and three clones ΔRNF4 complemented with chicken mCherry-Rnf4. **(b)** Wild type cells; ΔRNF4; ΔUBE2W and three clones ΔUBE2W complemented with Human Ube2w isoform2. **(c)** Wild type cells; ΔRNF4; ΔRNF4 ΔUBE2W and three clones ΔRNF4 ΔUBE2W complemented with Human Ube2w isoform2. Data represented as indicated: Wild type (Blue losange), ΔRNF4 (red square), ΔUBE2W (brown circle), ΔRNF4 ΔUBE2W (green triangle) rescue clone 1 (yellow triangle), rescue clone 2 (violet losange), rescue clone 3 (circle opal). Error bars represent 2 SD. **(d-f)** Whole cells extracts of chicken DT40 Wild type cells and rescue clones were analysed by western blotting using the indicated antibodies. **(g)** and **(h)** shows absence of complementation of ΔUBE2W and ΔRNF4 ΔUBE2W by Human Ube2w-EGFP fusion in colony survival assay as describe in (Fig. 1).

**
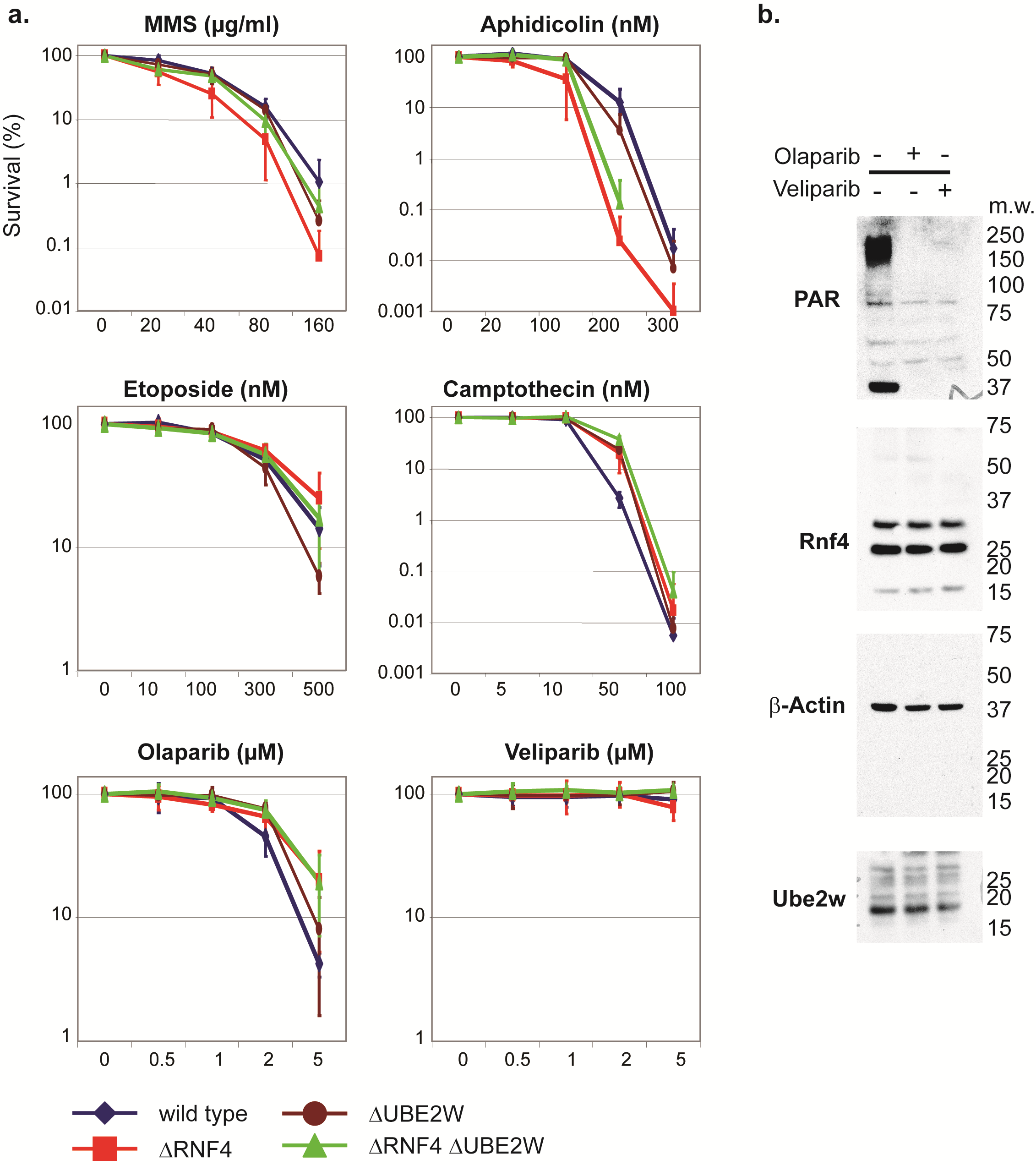
**

**Supplementary Figure 2: mutants sensitivity to DNA damage drug and PARP inhibitor**

**(a)** Wild type cells and cells deficient for ΔRNF4; ΔUBE2W and ΔRNF4, ΔUBE2W were subjected to DNA damaged induced stress by MMS, aphidicolin, etoposide, camptothecin, olaparib and veliparib as indicated. Mutants sensitivity was determined by colony formation assay as describe (Fig. 1). **(b)** Wild type DT40 cells were treated with anti PARP inhibitor olaparib or veliparib for 1H. Protein were analysed by western blotting using antibodies against PAR (top panel) and Rnf4, β-actin and Ube2w (bottom panel). Molecular weight marker is indicated on the right inside (kDa).

**
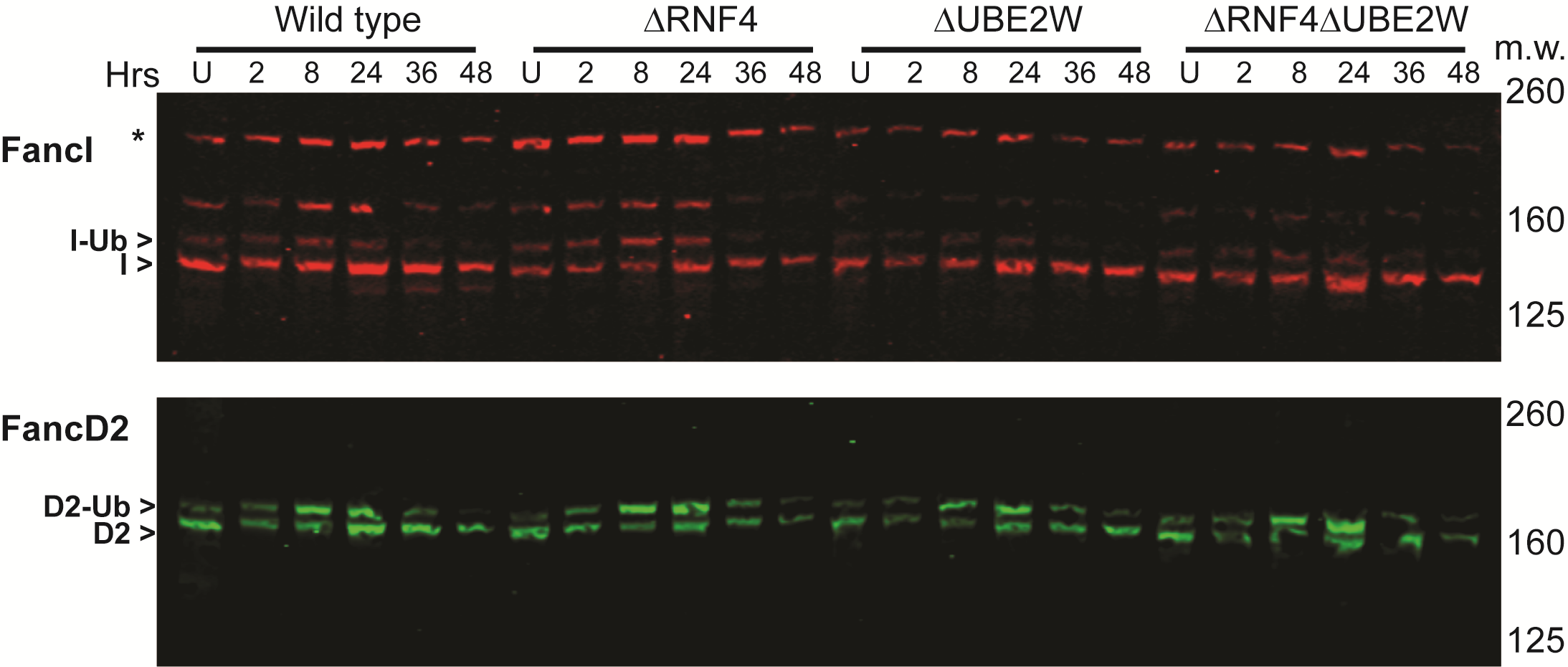
**

**Supplementary Figure 3: Increase and prolong FancD2/I monoubiquitination induced by MMC in ΔRNF4 is rescued by UBE2W inactivation observed by *LiCOR***

Wild type cells and cells deficient for ΔRNF4; ΔUBE2W and ΔRNF4 ΔUBE2W were treated with Mitomycin C (100ng/ml) for 1H (As describe Fig. 4). Protein were analysed by *LiCOR* using antibodies against FancI (top panel) and FancD2 (bottom panel). The recovery time after MMC treatment of each protein extraction is indicated on each lane of the top panel in hours. Non-ubiquitinated and mono-ubiquitinated form of FancI and FancD2 are indicated by an arrows head on the left inside (respectively: I >, D2 > and I-Ub >; D2-Ub >). Asterisk indicates non specific band (*). Molecular weight markers are indicated on the right inside (kDa). Quantification of the ratio of ubiquitinated protein to total protein has been determined by three independent LiCOR experiment can be seen in (Fig. 4).

**
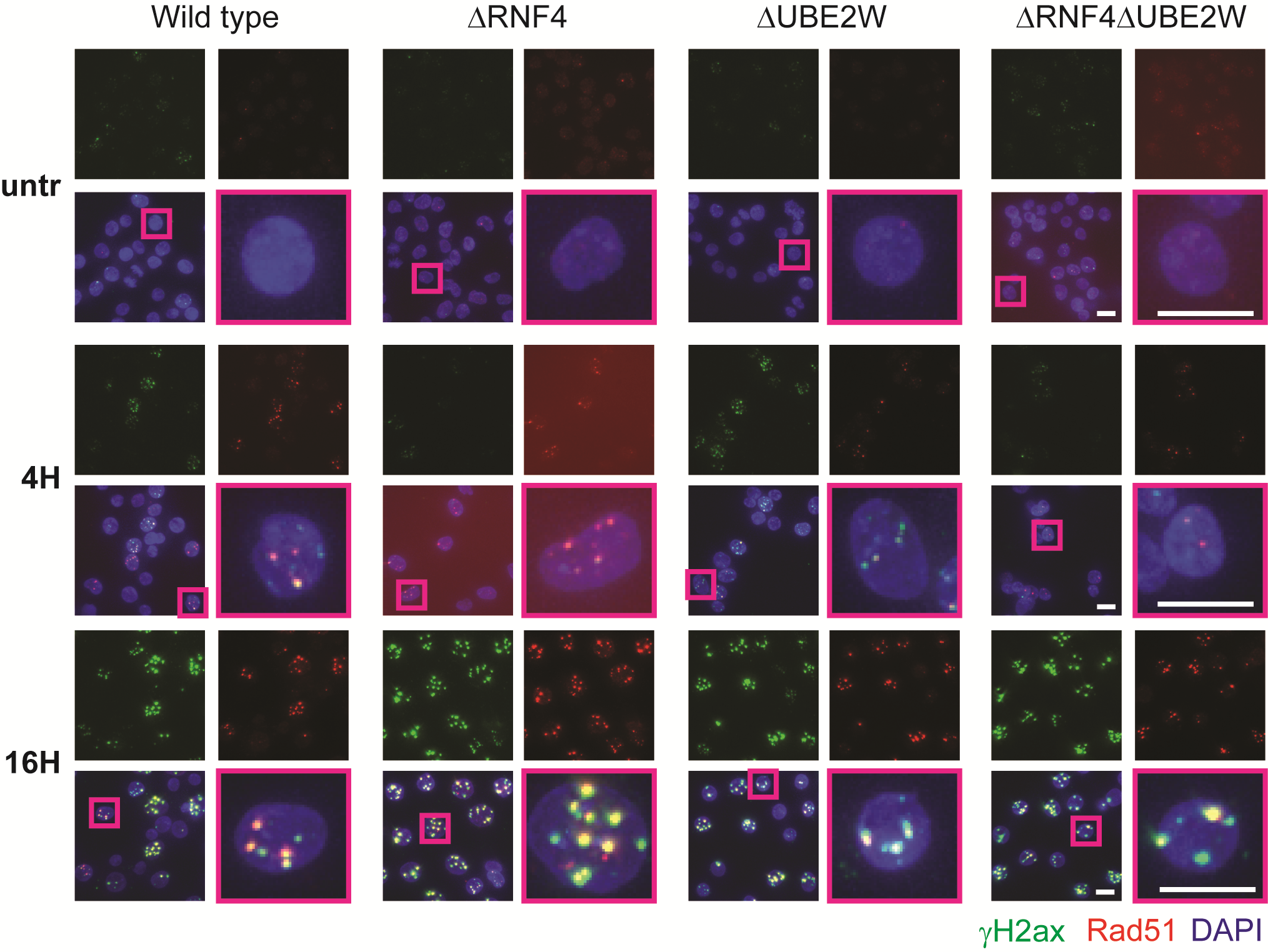
**

**Supplementary Figure 4: Prolonged DNA damage induced foci formation in ΔRNF4 cells is suppressed by UBE2W inactivation**

After treatments with or without MMC (50ng/ml) for 1H, cells were allowed to recover for 4H or 16H. For each indicated cell line, the formation of MMC induced foci for γ-H2ax (green) and Rad51 (red) was analysed by immunostaining. Chomatin was stained by DAPI (Blue). Panel of representative cells are shown fro each staining. Pink squares mark a representative cell which is shown magnified in each bottom right corner. White bar: 10 um. Staining of individual cells and foci quantification are shown in (Fig. 5).


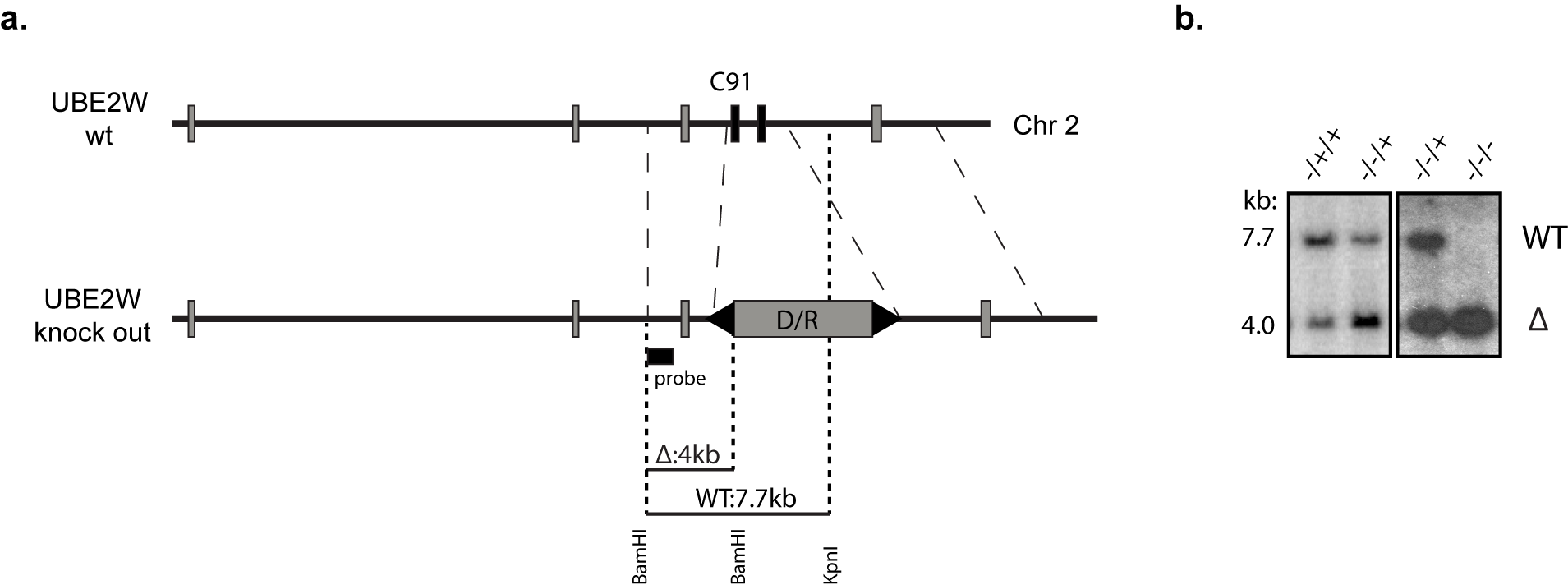


**Supplementary Figure 5: Generation of *UBE2W* disruption in DT40 cells.** **(a)** Schematic representation of the genomic locus of *UBE2W* on chromosome II with exon configuration. The position of the catalytic C91 and the gene disruption construct with exon 4 and 5 replaced by drug resistance cassettes (D/R) is indicated. **(b)** Southern blot analysis was performed on BamHI/KpnI-digested genomic DNA from heterozygous (-/+/+ and -/-/+) and homozygous (-/-/-) genotypes. The position of the probe used is indicated by the bar. wt: wild-type locus; ∆: gene knockout locus.

Supplementary Table 1 : Antibodies used

| Antibody | Used | dilution | species | Reference |
| --- | --- | --- | --- | --- |
| mAb414 | Wb | 5000 | Mouse | Covance (MMS-102P) |
| β-actin | Wb | 25 000 | Mouse | SIGMA |
| FancI | Wb/ LiCOR | 1000 | Rabbit | KJ Patel gift |
| FancD2 | Wb/ LiCOR | 3000 | Rabbit | KJ Patel gift |
| γ -H2ax | IF | 500 | Mouse | Upstate (05-636) |
| PAR | wb | 3000 | Rabbit | BD Pharminger (551813) |
| Rnf4 | Wb | 2000 | Sheep | This study |
| Rad51 | Wb/IF | 5000/ 100 | Rabbit | Santa Cruz (H-92) |
| SUMO1 | wb | 500 | Mouse | Zymed (33-2400) |
| SUMO2/3 | wb | 2000 | Rabbit | Zymed (51-9100) |
| Ube2w | Wb | 2000 | Sheep | This study |
| Vinculin | Wb | 5000 | Mouse | Abcam (ab1058) |
| Ubiquitin | wb | 2000 | Rabbit | DAKO (Z0458) |
| Mouse alexa488 | IF | 300 | Goat | Invitrogen (A11029) |
| Rabbit alexa568 | IF | 300 | Goat | Invitrogen (A11036) |
| Mouse-HRP | Wb | 3000 | Goat | SIGMA (A9917) |
| Rabbit-HRP | Wb | 3000 | Goat | SIGMA (A6154) |
| Sheep-HRP | Wb | 4000 | Donkey | SIGMA (A3415) |
| Rabbit-680LT | Li-COR | 25 000 | Donkey | LI-COR (926-68023) |
| Rabbit-800CW | Li-COR | 25 000 | Donkey | LI-COR (926-32213) |
